# Dapagliflozin Reduces Apoptosis of Diabetic Retina and Human Retinal Microvascular Endothelial Cells Through ERK1/2/cPLA2/AA/ROS Pathway Independent of Hypoglycemic

**DOI:** 10.3389/fphar.2022.827896

**Published:** 2022-02-24

**Authors:** Yuxin Hu, Qian Xu, Hongxue Li, Ziyu Meng, Ming Hao, Xuefei Ma, Wenjian Lin, Hongyu Kuang

**Affiliations:** The Department of Endocrinology, The First Affiliated Hospital of Harbin Medical University, Harbin, China

**Keywords:** dapagliflozin, diabetic retinopathy, Apoptosis, metabolomics, arachidonic acid

## Abstract

**Introduction:** It is known that the metabolic disorder caused by high glucose is one of pathogenesis in diabetic retinopathy (DR), the leading cause of blindness, due to the main pathological change of apoptosis of endothelial cells (ECs). In previous studies, the potential impact of sodium glucose cotransporter-2 (SGLT-2), whose inhibitors slow the progression of DR, has not been elucidated. The purpose of the presented study was to explore the effect of SGLT-2 inhibitors dapagliflozin (DAPA) on apoptosis of diabetic mice retina and human retinal microvascular endothelial cells (HRMECs), examine the effects of dapagliflozin on HRMECs metabolism, and explore the molecular processes that affect DR.

**Methods and Results:** The eyeballs of male streptozotocin (STZ)-induced diabetic C57BL/6N mice were evaluated. C57BL/6N mice were divided into control group (CON), diabetic untreated group (DM), diabetic dapagliflozin treatment group (DM + DAPA) and diabetic insulin treatment group (DM + INS). Hematoxylin-Eosin (HE) staining was performed to observe the pathological structure of the mice retina, and TUNEL staining to detect apoptosis of mice retinal cells. *In vitro*, DCFH-DA and western blot (WB) were used to evaluate ROS, Bcl-2, BAX, cleaved-caspase 3 in HRMECs and metabolomics detected the effect of dapagliflozin on the metabolism of HRMECs. And then, we performed correlation analysis and verification functions for significantly different metabolites. *In vivo*, dapagliflozin reduced the apoptosis of diabetic mice retina independently of hypoglycemic. *In vitro*, SGLT-2 protein was expressed on HRMECs. Dapagliflozin reduced the level of ROS caused by high glucose, decreased the expression of cleaved-caspase3 and the ratio of BAX/Bcl-2. Metabolomics results showed that dapagliflozin did not affect the intracellular glucose level. Compared with the high glucose group, dapagliflozin reduced the production of arachidonic acid (AA) and inhibited the phosphorylation of ERK1/2, therefore, reducing the phosphorylation of cPLA2, which is a key enzyme for arachidonic acid release.

**Conclusion:** Collectively, results unearthed for the first time that dapagliflozin reduced apoptosis of retina induced by DM whether *in vivo* or *in vitro*. Dapagliflozin did not affect the glucose uptake while mitigated intracellular arachidonic acid in HRMECs. Dapagliflozin alleviated HRMECs apoptosis induced by high glucose through ERK/1/2/cPLA2/AA/ROS pathway.

## Introduction

Diabetes mellitus (DM) is one of the most serious health problems in the present world (Zimmet et al., 2016). More than one-third of people with diabetes have signs of diabetic retinopathy (DR), and the increasing prevalence of DM suggests that there will be more people with DR in the future (Yau et al., 2012). Devastating microvascular complications caused by DR can lead to blindness and affect the overall quality of life (Cole and Florez, 2020), and thus, preventing/retarding the progress of DR is an important concern in the treatment of DM.

Many experiments on the metabolism and mechanism of DR have been attempted and hyperglycemia and dyslipidemia are considered the most influential contributors among a variety of systemic factors with long-term effects (Yu Y, 2005). Studies have shown that there are significant metabolic abnormalities in DR (Razquin et al., 2018; Lu et al., 2019). High glucose disrupts intracellular glucose homeostasis and breaks the balance of metabolic enzymes, intermediate metabolites, and end products (Mesquida et al., 2019). The metabolic disorders resulting from high glucose lead to the activation of polyol pathway and hexosamine pathway, the accumulation of advanced glycation end-product, the enhancement of protein kinase C activity, and ultimately the generation of oxygen free radicals (M, 2001; RA., 2005; Wong et al., 2016; Forrester et al., 2020), which further promotes mitochondrial damage, inflammation, apoptosis and tissue damage (Kowluru and Mishra, 2015; Mesquida et al., 2019). Despite many experimental on the metabolism and mechanism of DR, some aspects remain to be clarified in detail, such as the effects of drugs on DR metabolism.

In a high glucose environment, a series of negative results occur including pericytes loss, apoptosis and proliferation of endothelial cells (ECs), disruption of blood-retinal barrier (BRB), and retinal neovascularization in retinal tissue (Wan et al., 2021), among which ECs play an indispensable role in maintaining the BRB and are the first cells to sense blood glucose changes (Kim et al., 2019; Yang et al., 2020). Activated by high glucose, many of the pathways mentioned above lead to metabolic disorders and the burden of oxidative stress in ECs (Elmasry et al., 2019), which are considered to be the main pathogenesis of DR (RA. et al., 2001; Kanwar et al., 2007). The accumulation of reactive oxygen species (ROS) caused by metabolic disorders increases the migration of retinal vascular endothelial cells, motivates the apoptosis of cells, changes the permeability of retinal vascular, and adds the leakage of basement membrane in the retina (Hsu et al., 2020). Hence, a possible therapeutic target to delay the progression of DR is to improve ECs function under a high glucose condition.

Dapagliflozin (DAPA) is a sodium glucose cotransporter-2 inhibitor (SGLT-2i) (Mudaliar et al., 2015). It serves as a novel antihyperglycemic agent by selectively blocking renal glucose reabsorption and thereby facilitating the elimination of blood glucose into the urine (Ferrannini, 2017). SGLT-2 inhibitors can not only lower blood glucose but also induce body weight, blood pressure, and circulating lipid levels. In addition, SGLT-2 inhibitors have been found to have protective effects on the heart, kidneys, and retinas (Fitchett et al., 2016; Neal et al., 2017; Cho et al., 2018; Mishriky et al., 2018; Wiviott et al., 2019). Studies have shown that DAPA protects the heart and kidneys by reducing the apoptosis of cells (Shih et al., 2021; Xing et al., 2021). Recent reports have also shown that SGLT-2 inhibitors reduced macrovascular and microvascular complications by affecting vascular remodeling (Dziuba et al., 2014; Ott et al., 2017). And according to Cho et al., SGLT2i slowed the progression of DR in patients with type 2 diabetes compared with those treated with sulfonylureas, and the benefit was independent of hypoglycemia (Cho et al., 2018). Nevertheless, a detailed understanding of how DAPA reverses high glucose-induced retinal endothelium dysfunction is lacking.

Metabolomics has emerged as one of the “omics” techniques and a powerful method for supervising different physiological and pathological procedures and responses to all kinds of therapeutic interventions (Nicholson et al., 1999). A study of DAPA on plasma metabolomics of patients with diabetic nephropathy suggests that DAPA may have protective effects on the kidney by revising molecular processes associated with energy metabolism, mitochondrial function, and endothelial function (Mulder et al., 2020). Consequently, metabolomics would assist in finding new therapies to reduce human suffering, bring innovative ideas to predict the efficacy and long-term benefits of diabetic retinopathy and its treatment strategies (Bugáňová. et al., 2017).

Therefore, we hypothesized that DAPA could suppress apoptosis of diabetic mice retina and HRMECs caused by high glucose. The effect of DAPA on HRMECs metabolism was studied by metabolomics, and the possible mechanism of DAPA reducing apoptosis was explored.

## Materials and Methods

### Experimental Animals and Groups

Twenty C57BL/6N mice (male, 6 weeks old, weighting 25–30 g) were obtained from Beijing Vital River Laboratory Animal Technology Co. Ltd (Beijing, China). General feeding for a week, mice were randomized into two groups: the control group (CON, *n* = 5), fed with regular chow diet, and the model group (*n* = 15), fed with high-fat feed (HFD). To generate the STZ-induced model of DM, mice in the model group were intraperitoneally injected with STZ (30 mg/kg in 50 mM citrate buffer, pH 4.5) after 4 h of fasting; for control, mice in the control group were given the same volume of citrate buffer. One week after STZ injection, the mice were considered DM if their blood glucose levels were greater than 300 mg/dl. Four weeks after the injection, the model group was randomly assigned to three groups: diabetes untreated group (DM, n = 5), dapagliflozin treatment group (DM + DAPA, *n* = 5, 10 mg/kg, gavage), and insulin treatment group (DM + INS, *n* = 5, 0.1IU/kg, intraperitoneal injection); the control group and the untreated group were given the same dose of normal saline by gavage once a day. The blood glucose and hemoglobin A1c (HbA1c) of the mice were measured. After 12 weeks of treatment, mice were sacrificed anesthetized and eyeballs were immediately extracted. The animal experiments were approved by the Ethics Committee of The First Affiliated Hospital of Harbin Medical University.

### Cell Culture and Treatment

Human retinal microvascular endothelial cells (HRMECs) were obtained from the Beijing Beina Chuanglian Institute of Biotechnology (Beijing, China). Cells were cultured in DMEM including 10% fetal bovine serum (FBS) and 1% antibiotics (100 U/mL penicillin and 100 mg/ml streptomycin). The cell culture environment was a humidified atmosphere of 5% CO_2_ at 37°C. HRMECs in the logarithmic growth phase were used for experiments. There were four stages of cell experiments. In the first stage, HRMECs were incubated with different glucose concentrations (5.5 mmol/L, 30 mmol/L, 60 mmol/L) for 24 h. In the second stage, HRMECs were incubated with high glucose (30 mmol/L) with or without DAPA (1 μmol/L) for 24 h. In the third stage, HRMECs were incubated with different concentrations of 2-(N-(7-nitrobenz-2-oxa-1, 3-diazol -4-yl) amino)-2-deoxy-D-glucose (2-NBDG, Invitrogen, United States) (0, 50, 100, 200, 300, 400, 500, 600, 700, 800, 900, 1000 μmol/L) for 30 min or with 2-NBDG for different times (0, 5, 15, 30, 60, 120 min) with or without DAPA (1 μmol/L). In the fourth stage, HRMECs were incubated with different glucose concentrations (5.5 mmol/L, 30 mmol/L) with or without DAPA (1 μmol/L) for 24 h.

### Hematoxylin-Eosin Staining

The eyeballs were immediately separated and fixed in the eye fixative solution. Sections of retinal tissue were embedded in paraffin, stained with hematoxylin and eosin, dehydrated with ethanol and xylene, and then sealed with neutral glue. Images were scanned by using AperioCS2 Leica.

### TUNEL Staining

TUNEL staining was conducted using the ApopTag kit (Oncor, Purchase, NY, United States). Paraffin sections were dewaxed and repaired with proteinase K and then incubated with TdT enzyme and d UTP for 2 h at 37°C while nuclei were stained with DAPI. Images were scanned and analyzed using Aperio CS2, Leica.

### Western Blotting

RIPA buffer containing 10% phosphatase inhibitor (Beyotime, China) and 1% protease inhibitor (Beyotime, China) was used to lysate HRMECs and obtain proteins. The extracted protein concentration was measured using the BCA protein concentration assay kit (Beyotime, China). Protein samples of equal quality were resolved on 10% or 12% SDS-PAGE, then transferred to PVDF membranes, blocking the membrane with 5% skim milk at room temperature for 1 h, and the membranes were incubated at 4°C overnight with rabbit polyclonal antibody to SGLT-2 (Abcam, United States), BAX, Bcl-2, cleaved-caspase-3, ERK1/2, phosphorylated-ERK1/2, cPLA2 (Wanleibio, China), and rabbit polyclonal antibody to phosphorylated-cPLA2 (Cell Signaling Technology, United States). Mouse monoclonal antibodies to GAPDH (Abcam, United States) and β-actin (Abmart, China) were used as control. The membranes were incubated with the fluorescent secondary antibody (IRDye 800 CW goat anti-rabbit, 926-32211 or IRDye 680RD goat anti-mouse, 926-68070, 1:10000, LI-COR) at room temperature, dark for 1 h. The protein bands were scanned by using Odyssey Infrared Laser Scan-imaging Instrument (LI-COR). ImageJ was used for images analysis. All experiments were repeated three times.

### RNA Extraction and Gene Expression Analysis

Firstly, total RNA was extracted using an RNA isolation kit (Axygen, United States). Secondly, RNA was reverse-transcribed into cDNA using a ReverTra Ace qPCR-RT Master mix kit (Toyobo, Japan). Then, the cDNA was analyzed using SYBR Green qPCR Mix (Toyobo, Japan). qPCR was performed with a 7,500 Real-time PCR system (Applied Biosystems, United States). Target RNA expression was calculated using the 2-∆∆Ct comparative method and normalized to internal standards [glyceraldehyde-3-phosphate dehydrogenase (GAPDH)]. The primers sequence of SGLT-2 (human) were as follows: forward, 5′-CTG​TTT​GCA​CCC​GTG​TAC​CT-3’; reverse, 5′-CCT​GTC​ACC​GTG​TAA​ATC​AT GG-3’. The sequences of primers used to amplify GAPDH were as follows: forward, 5′-ACA​ACT​TTG​GTA​TCG​TGG​AAG​G-3; reverse, 5′-GCC​ATC​ACG​CCA​CAG​TTT​C-3’. All experiments were repeated three times.

### ROS Measurement

The fluorescent probe 2′,7′-dichlorofluorescin diacetate (DCFH-DA; Beyotime, China) were used to determine cellular reactive oxygen species (ROS) levels. Cells were incubated with 10 mmol/L DCFH-DA at 37°C in the dark for 25 min s. DCFH-DA was removed and cells were washed rinsed with serum-free DMEM. The fluorescence of intracellular ROS was observed using electron fluorescence microscopy. The fluorescence intensity was used to illustrate the ROS generated under different treatment conditions. The levels of fluorescence product were assessed using a fluorescent microplate reader, the excitation wavelength was 488 nm. All experiments were repeated three times.

### Non-Targeted Metabolomics

There were 2 groups to be tested: high glucose (HG, 30 mmol/L) or high glucose (30 mmol/L) with DAPA (1 μmol/L) (HG + DAPA). Each group had 6 biological replicates. HRMECs were washed three times with cold PBS and scraped from the dish. After centrifugation (6,000 g for 5 min at 4°C), HRMECs containing the metabolites were kept at −80 °C until measurement. The samples were thawed at 4°C, added with 200 μL pre-cooled d water and 800 μL pre-cooled methanol/acetonitrile (1:1, v/v), ultrasonically pulverized in an ice bath for 1 h, incubated at -20°C for 1 h to deposit protein, centrifuged 16,000 g for 20 min at 4°C, and then separated the supernatant. The supernatant was redissolved by adding 100 μL of acetonitrile water (1:1, v/v), and centrifuged at 16,000 g for 20 min at 4°C again. The stability and performance of the machine were checked by inserting a quality control (QC) sample into the test sample The samples were separated using an ultra-high performance liquid chromatography system (UHPLC). Metabolites were analyzed using a Triple-TOF 5,600 mass spectrometer (AB SCIEX). Principal components analysis (PCA), partial least-squares discriminant analysis (PLS-DA), and orthogonal partial least-squares (OPLS) analysis were performed on normalized data using SIMCA-P 14.1 software (Umetrics, Sweden). The screening criteria for differential metabolites between the two groups were both VIP values (VIP >1) and *t*-test (*p* < 0.05). Metabolites were identified from the online databases: KEGG (http://www.kegg.jp). Pathway analysis was performed using MetaboAnalyst. An impact value >0.1was used to determine the most relevant pathways.

### Glucose Uptake

Glucose uptake in HRMECs was tested by2-NBDG. Different concentrations of 2-NBDG (0, 50, 100, 200, 300, 400, 500, 600, 700, 800, 900, 1000 μmol/L) or different times (0, 5, 15, 30, 60, 120 min) with or without dapagliflozin (1 μmol/L) were used to treat HRMECs. After incubating at 37°C for 30 min, HRMECs were washed three times with KRB (Krebs-Ringer’s HEPES [KRH] buffer). Intracellular fluorescence quantification and images of 2-NBDG were analyzed at 488 and 535 nm emission using a multifunctional microplate and electron fluorescence microscope (Molecular Devices, United States). All experiments were repeated three times.

### ELISA

Intracellular arachidonic acid (AA) levels were determined using ELISA kits (Elabscience Biotechnology, China). All experiments were repeated three times.

### Statistical Analysis

Statistical analysis was carried out using GraphPad PRISM. Data were presented as means ± SD. An unpaired *t*-test was used for comparison between the two groups. The differences were considered statistically significant when *p* < 0.05.

## Results

### Dapagliflozin Mitigated Diabetes-Induced Retina Apoptosis Independent of Hypoglycemic

**FIGURE 1:**
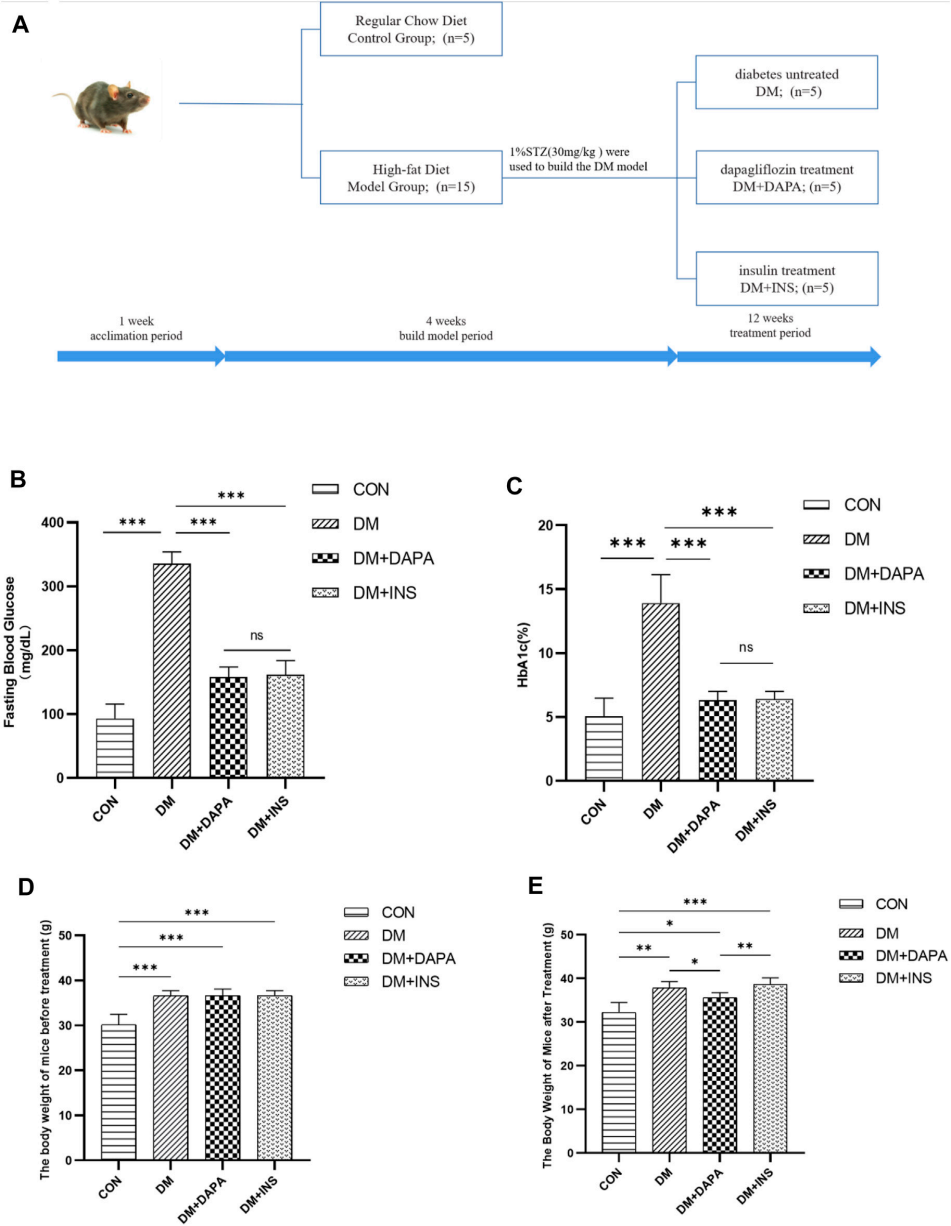
**(A)** Schematic diagram of experiment grouping. **(B,C)** Effect of dapagliflozin on blood glucose and HbA1c in diabetic mice. **(D,E)** Effect of dapagliflozin on body weight of mice Data are presented as means ± SD. *p < 0.05, **p < 0.01, ***p < 0.001.

**FIGURE 2:**
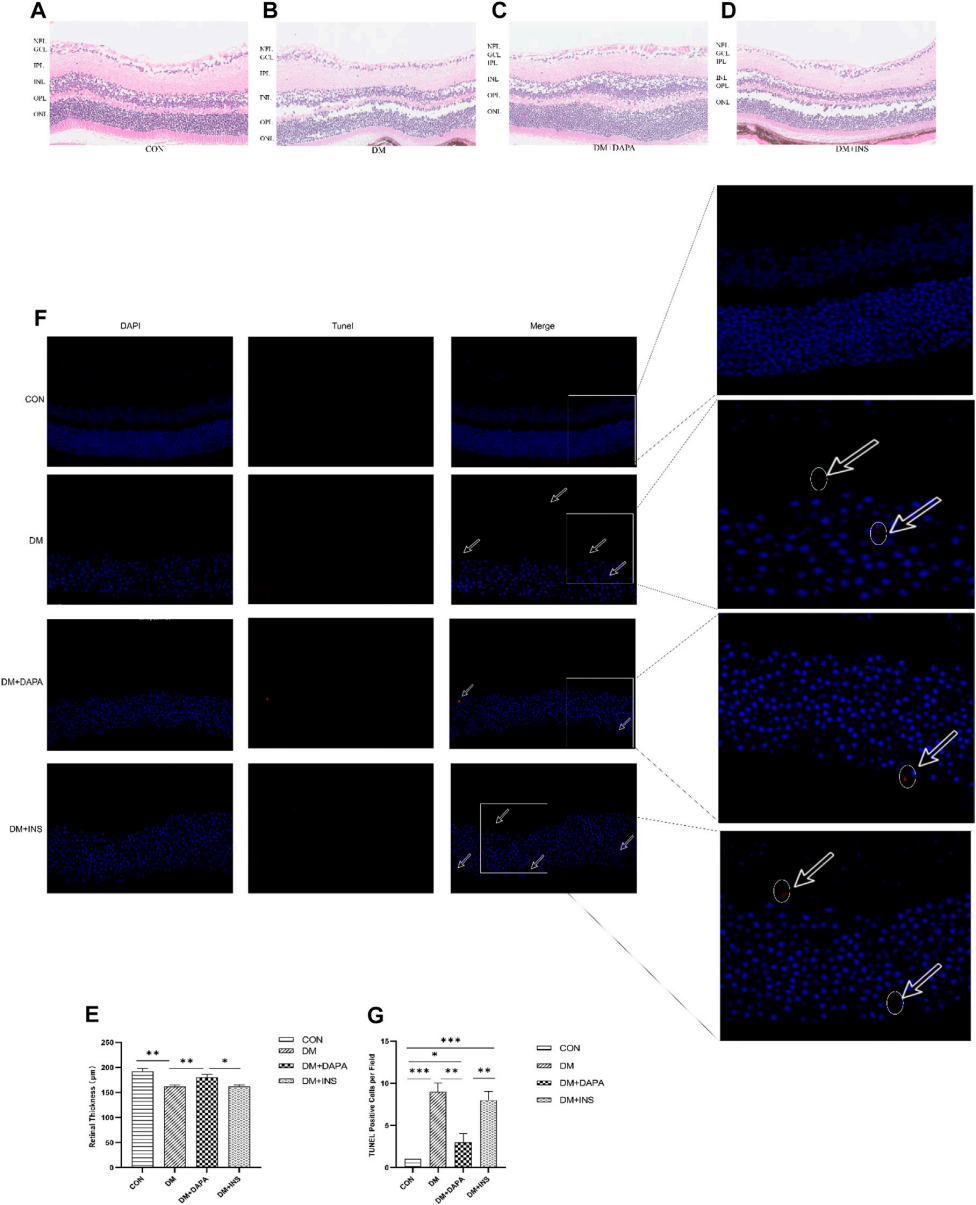
Effect of DAPA on diabetic mice retinal apoptosis. **(A–E)** H&E staining and retinal thickness analysis of mice retinal tissue. Scale bar: 250 μm. NFL: nerve fiber layer. GCL: ganglion cell layer. IPL: inner plexiform layer. INL: inner nuclear layer. OPL: outer plexiform layer. ONL: outer nuclear layer. **(F,G)** TUNEL staining image of mice retinal tissue and percentage of TUNEL positive cells. Scale bar: 50 μm. Data are presented as means ± SD. *p < 0.05, **p < 0.01, ***p < 0.001.

After successful modeling, the mice were divided into four groups: the control group (CON), the diabetes mellitus group (DM), the dapagliflozin treatment group (DM + DAPA), and the insulin treatment group (DM + INS) **(** Figure 1A). Mice that were treated with either DAPA or insulin showed a marked reduction in hyperglycemia and HbA1c. There was no difference of DAPA or insulin on reducing blood glucose and HbA1c in diabetic mice. **(** Figures 1B,C). Before treatment, the body weight of the DM group, DM + DAPA group, and DM + INS group was higher than that of the CON group, but there was no significant difference in body weight among the three groups **(** Figure 1D). After treatment, compared with the CON group, the body weight of mice in the DM + DAPA group decreased, but no difference in the DM + INS group **(** Figure 1E). We examined the retina in each group by H&E staining and found that retinas in the CON group were well-structured and tightly arranged whereas retinal cells in the DM group were less, disordered and the intercellular space increased. Interestingly, in diabetic mice treated with DAPA, retinal cells were arranged in a relatively clean and intact manner, but the retina structure was not improved after insulin treatment **(** Figures 2A–D). Likewise, H&E staining also illustrated a reduction in retinal thickness due to diabetes, and DAPA attenuated such morphological changes **(** Figure 2E). To confirm whether DAPA alleviates retinal degeneration by reducing retinal apoptosis, TUNEL staining was performed, revealing that apoptotic cells were more prevalent in DM, while DAPA-treated mice showed a significant reduction in the number of TUNEL-positive cells, which was not observed in the insulin-treated group **(** Figures 2F,G **)**.

### SGLT-2 Was Expressed on HRMECs and Dapagliflozin Mitigated High Glucose–Induced ROS and Apoptosis in Cultured HRMECs

**FIGURE 3:**
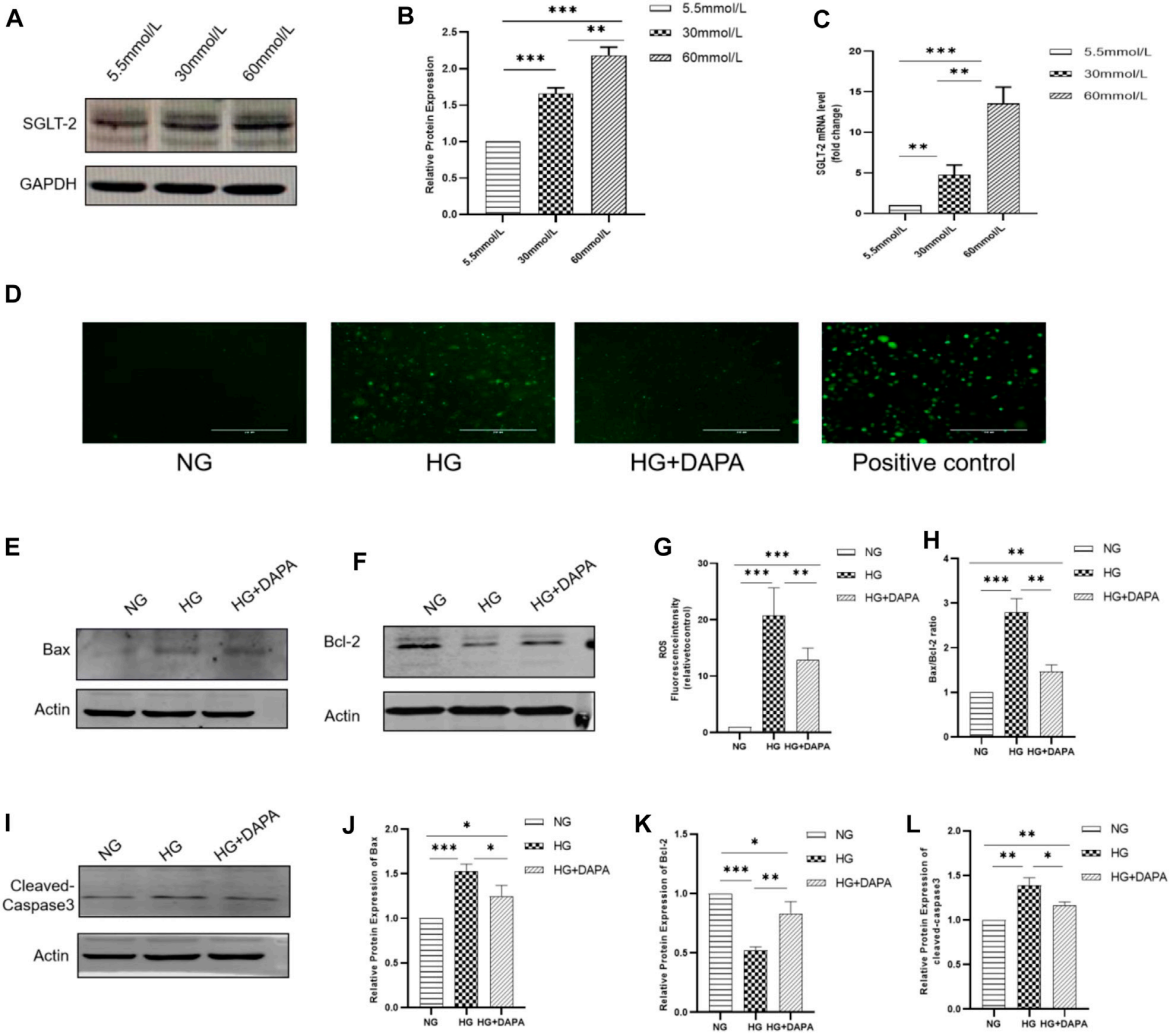
Expression of SGLT-2 in HRMECs and effect of DAPA on ROS and apoptosis of HRMECs. **(A,B)** Determination of SGLT-2 expression in HMRECs by WB. **(C)** Determination of SGLT-2 mRNA expression in HMRECs by RT-PCR. **(D,G)** The effect of DAPA on the production of ROS. **(E,F,H,I–L)** The protein expression of BAX, Bcl-2, and cleaved-caspase-3 was evaluated by WB. Data are presented as means ± SD. *p < 0.05, **p < 0.01, ***p < 0.001.

HRMECs were incubated with different glucose concentration (5.5 mmol/L, 30 mmol/L, 60 mmol/L) for 24 h. WB and qPCR were used to determine the expression of SGLT-2 in HRMECs. Our study found that SGLT-2 was expressed on HRMECs. And with the glucose concentration increased, the expression of SGLT-2 increased **(** Figures 3A–C). The results showed that when the glucose concentrations were 30 mmol/L and 60 mmol/L, the level of SGLT-2 protein increased by 1.66 times and 2.18 times, and the level of SGLT-2 mRNA increased by 4.68 times and 13.56 times, respectively, in comparison with that of 5.5 mmol/L. To further explore the effect of DAPA on ROS and apoptosis, DCFH-DA was used to measure the level of ROS, and the expression of BAX, Bcl-2, and cleaved-caspase-3 of HRMECs was determined by WB. Our research showed that compared with the HG group, the cells incubated with DAPA reduced the level of ROS caused by high glucose **(** Figures 3D,G). HG increased intracellular ROS levels by approximately 23.73-fold, while DAPA reduced this change to 12.83-fold. At the same time, HRMECs showed a significant up-regulation of apoptosis-associated proteins, containing BAX, cleaved-caspase-3, and down-regulation of anti-apoptosis associated proteins Bcl-2 after incubation with high glucose. While treatment with DAPA rescued the changes of these proteins **(** Figures 3E,F,H, I–L **)**.

### Dapagliflozin Affected Metabolites in HRMECs

**FIGURE 4:**
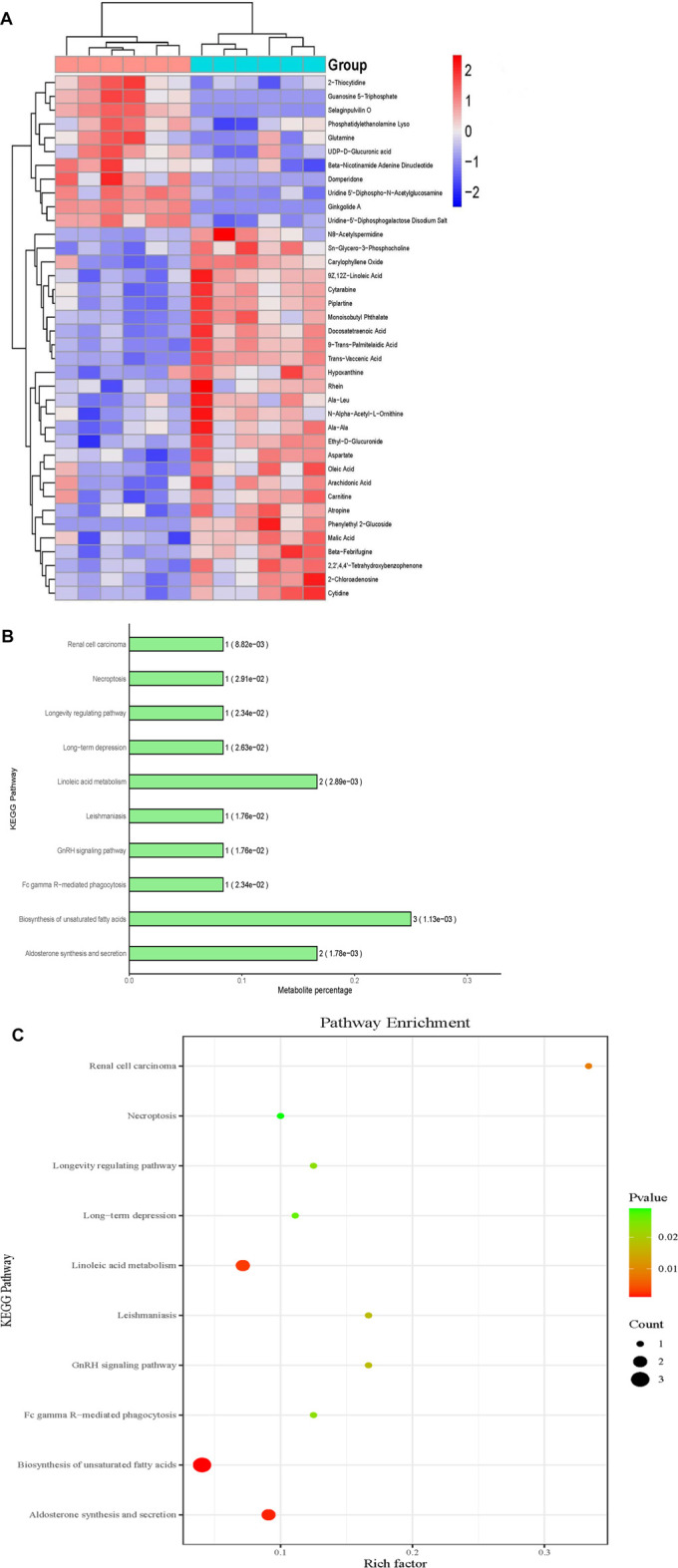
Effects of DAPA on the metabolites of HRMECs. **(A)** Metabolites hierarchical clustering analysis. Blue means down-regulation, and red means up-regulation. The darker the color, the more obvious the difference. **(B)** Bar chart of significantly different metabolites KEGG metabolic pathway enrichment analysis (Top10). **(C)** Bubble chart of significantly different metabolites KEGG metabolic pathway enrichment analysis (Top10). The abscissa represented the enrichment rate; the larger the graph, the greater number of differential metabolites; the color indicated the significance of enrichment, namely *p*-value, and the redder indicated the more significance, the color gradient on the right indicated the size of the *p*-value.

HRMECs were incubated in HG with DAPA for 24 h or not. To comprehensively and intuitively demonstrate the relationship between samples and the differences in the expression patterns between the two groups, we used qualitatively significant differences in the expression of metabolites to perform hierarchical clustering for each group of samples **(** Figure 4A). The results showed that there were 38 significantly different metabolites in the two groups. Compared with the HG group, the levels of 11 metabolites were up-regulated and 27 metabolites were down-regulated in HG with DAPA group. We conducted KEGG ID Mapping on the significantly different metabolites obtained from the two groups and presented them to http://www.kegg.jp for relevant pathway analysis. The metabolites involved 20 statistically significant KEGG signaling pathways between the two groups. The enrichment results of metabolic pathways (TOP10) were represented by enrichment bar graphs and enrichment bubble graphs **(** Figures 4B,C).

### Dapagliflozin Mitigated High Glucose-Induced Intracellular Arachidonic Acid in HRMECs and Does Not Affect Glucose Uptake

**FIGURE 5:**
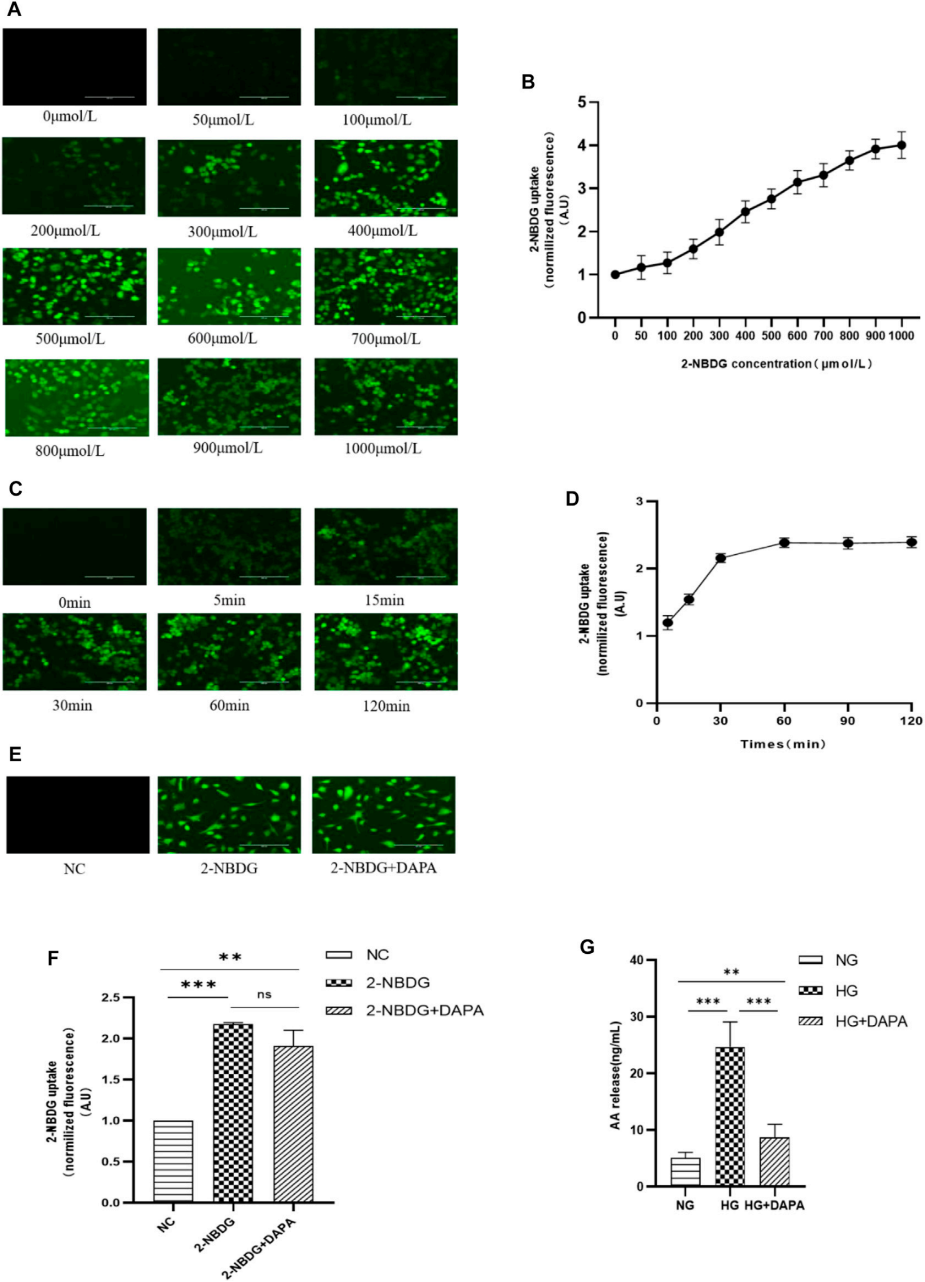
Effect of DAPA on glucose uptake and arachidonic acid (AA). **(A,B)** The concentration of 2-NBDG acting on HRMECs. **(C,D)** The time of 2-NBDG acting on HRMECs. **(E,F)** The effect of DAPA on glucose uptake of HRMECs. **(G)** The effect of DAPA on AA of HRMECs. Data are presented as means ± SD. *p < 0.05, **p < 0.01, ***p < 0.001.

According to the results of the above metabolomics, DAPA incubation reduced intracellular arachidonic acid (AA) caused by high glucose, while did not affect intracellular glucose **(** Figure 4A). We further measured the effects of DAPA on glucose uptake and AA using 2-NBDG and ELISA kits. After treating HRMECs with different concentrations and times of 2-NBDG, the fluorescence intensity in HRMECs was measured by a microplate reader and observed under an electron fluorescence microscope to obtain the optimal concentration and time for 2-NBDG to incubate HRMECs. The results showed that with the increase of 2-NBDG concentration and incubation time, the intracellular fluorescence intensity gradually increased **(** Figures 5A–D). Finally, the concentration and time of 2-NBDG incubated HRMECs that we chose at 400 μmol/L and 30 min for subsequent experiments. HRMECs were incubated in 2-NBDG (0 μmol/L, NC), 2-NBDG (400 μmol/L), and 2-NBDG (400 μmol/L) with DAPA (1 μmol/L) for 30 min. The results showed that DAPA could not affect the uptake of 2-NBDG by HRMECs which was consistent with the metabolomics results **(** Figures 5E, 4F). ELISA kits were used to measure intracellular AA. HRMECs were cultured in the normal glucose (NG, 5.5 mmol/L), the high glucose (HG, 30 mmol/L), the high glucose (HG, 30 mmol/L) with DAPA (1 μmol/L) (HG + DAPA). Our results showed that the concentration of AA in the HG group was significantly higher than that in the NG group. The concentration of the NG group was 5.659 ng/ml, while that of the HG group was 24.673 ng/ml. After DAPA intervention, the AA level decreased to 8.176 ng/ml. **(** Figure 5G). The results were consistent with metabolomics that DAPA reduced AA in HRMECs.

### Dapagliflozin Attenuated High Glucose-Triggered Activity of ERK1/2/cPLA2

**FIGURE 6:**
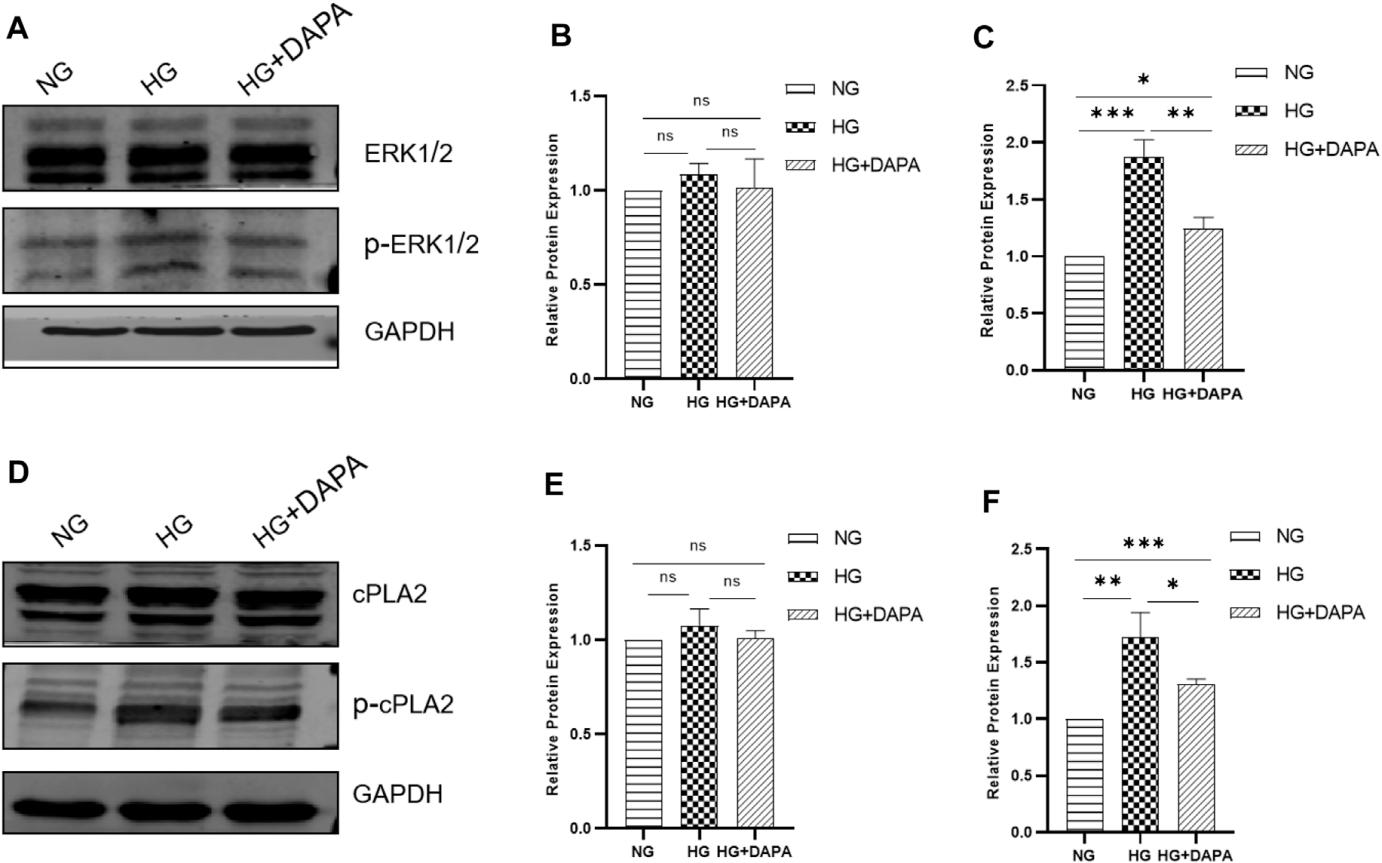
Effect of DAPA on Activity of ERK1/2/cPLA2. **(A–C)** Determination of the effect of DAPA on the activity of ERK1/2 and p-ERK1/2 in HMRECs by WB. **(D–F)** Determination of the effect of DAPA on the activity of cPLA2 and p-cPLA2 in HMRECs by WB. Data are presented as means ± SD. *p < 0.05, **p < 0.01, ***p < 0.001.

In investigating the mechanism of DAPA reducing arachidonic acid, we determined the enzymes phosphorylated ERK1/2 and cPLA2 that affect the release of arachidonic acid. HRMECs treated with the normal glucose (NG, 5.5 mmol/L), the high glucose (HG, 30 mmol/L), the high glucose (HG, 30 mmol/L) with DAPA (1 μmol/L) (HG + DAPA). After intervention for 24 h, expression of ERK1/2, cPLA2, and phosphorylated-ERK1/2, phosphorylated-cPLA2 were measured in protein level. Our research found that ERK1/2 and cPLA2 did not differ between the three groups. The phosphorylation of ERK1/2 and cPLA2 was increased when HRMECs were incubated with high glucose compared with the normal group, while DAPA reversed these changes **(** Figures 6A–F).

## Discussion

DR caused by chronic hyperglycemia is the most widespread microvascular complication of DM and is the leading cause of recognized acquired blindness of working age, endangering the health and life of DM patients. (Yau et al., 2012). Clinically common targeted treatment strategies for DR mainly include physical therapy based on laser photocoagulation therapy and drug therapy based on VEGF antibody, but these treatment methods have limitations. Therefore, the discovery of new targets and the development of new therapeutic drugs urgently need to be resolved.

DAPA, as a new type of hypoglycemic drug, is widely used in treatments of patients with diabetes. Studies have found that DAPA has protective effects on diabetic complications and other diseases by suppressing apoptosis (Jaikumkao et al., 2018; Shih et al., 2021). Preclinical studies have shown that SGLT-2 inhibitors alleviate dysfunction of the vascular, and this mechanism of action appears to be independent of the hypoglycemic effect (Alshnbari et al., 2020). Furthermore, one study has shown that DAPA reduced renal tubule damage and apoptosis in IR-injured mice (Chang et al., 2016). Consistent with the above findings, our results found that DAPA could reduce retinal tissue apoptosis in diabetic mice, and this effect was independent of the hypoglycemic effect.

Apoptosis of ECs possessed an important role in the pathogenesis of DR. The imbalance between proapoptotic and antiapoptotic factors is the key cause that triggers the initiation of its apoptotic program (Yang et al., 2020). To further understand the role of DAPA at the cellular level, we measured the expression of SGLT-2 on HRMECs and incubated HRMECs with DAPA to observe its effect on apoptosis caused by high glucose. According to studies, the possible mechanism of Bcl2 and BAX inhibiting apoptosis is that Bcl2 up-regulation can prevent BAX translocation and reduce mitochondrial damage. (Jaikumkao et al., 2018; Shih et al., 2021). Our experiment found that SGLT-2 was expressed on HRMECs for the first time, and DAPA dampened the retina apoptosis via lowering the c-caspase-3 expression, and the ratio of BAX/Bcl-2. And studies have found that DAPA can reduce the production of ROS in the cardiomyocytes and renal tubular epithelial cells *in vitro*, thereby reducing apoptosis (Jaikumkao et al., 2018; Shih et al., 2021). Our experiment presented that DAPA played the same role in reducing ROS-mediated apoptosis in HRMECs. In DM, exposure of cells to hyperglycemia leads to glucose autoxidation and free radical production (RA., 2005; Wong et al., 2016) Antioxidant and oxidative imbalance cause intracellular oxidative stress and ROS accumulation (RA., 2005). Oxidative modification of intracellular proteins, lipids, and DNA caused by high glucose further promotes mitochondrial damage, inflammation, apoptosis, and tissue damage (Mesquida et al., 2019). Therefore, consistent with other studies, DAPA reduced the production of ROS, thereby reducing HRMECs apoptosis.

According to metabolomics, there were 38 significantly different metabolites in the two groups. The metabolites involved 20 statistically significant KEGG signaling pathways between the two groups. The top ten were biosynthesis of unsaturated fatty acids, aldosterone synthesis and secretion, Linoleic acid metabolism, Renal cell carcinoma, GnRH signaling pathway, leishmaniasis, Fc gamma R-mediated phagocytosis, longevity regulating pathway, long-term depression, and necroptosis. Surprisingly, metabolomics results showed that DAPA did not affect glucose concentration in HRMECs. We used 2-NBDG to further verify the effect of DAPA on glucose uptake by HRMECs. The results showed that DAPA does not affect the fluorescence intensity of 2-NBDG in HRMECs, which is consistent with the results of metabolomics. Glucose transporter 1 (GLUT-1) is the major glucose transporter protein on retinal endothelial cells. (Choi, 2017). In addition, chronic hyperglycemia increases the expression of GLUT1 in the inner blood-retinal barrier of diabetic patients. (A K Kumagai, 1996; Veys et al., 2020). We hold the opinion that HRMECs express SGLT-2, while their ability to transport glucose may be weak, or there may be a compensatory effect of GLUT-1.

On the other hand, the results of metabolomics provided important information that DAPA can reduce the production of arachidonic acid (AA) caused by high glucose. Our data revealed that the most likely pathway for DAPA to reduce apoptosis was AA/ROS/apoptosis, since DAPA caused the reduction of intracellular arachidonic acid, resulting in the reduction of ROS production, and thus the reduction of cell apoptosis. AA is an essential polyunsaturated fatty acid. (Cao, 2000), and the main enzymes in AA metabolism include Cyclooxygenase (COX), lipoxygenase (LOX), and cytochrome P450 (CYP) enzymes. Under the catalysis of these enzymes, AA is metabolized to eicosanoids, which are involved in the progression of DR (Wang et al., 2020). Eicosanoids cause the accumulation of ROS in cells, which in turn causes cell apoptosis. Research has emphasized the significance of COX-derived metabolites, LOX-derived eicosanoids, CYP-derived epoxyeicosatrienoic acids to ROS, and retinal microvascular abnormalities (Zangar et al., 2004; Cao et al., 2012; Othman et al., 2013).

Hyperglycemia activates Phospholipase A2s (PLA2s), a family of enzymes that catalyze and release free fatty acids from glycerophospholipids, including AA and lysophospholipids (Han et al., 2003). Cyto-plasma lipase A2 (cPLA2) is a key enzyme that mobilizes AA from membrane phospholipids to release into the cytoplasm. Our results found that DAPA reduced the phosphorylation of cPLA2. Studies have shown that cPLA2 is phosphorylated in the model of diabetic retinopathy and retinal endothelial cells, which promote the release of AA from the cell membrane, causing retinal endothelial cells injury and death, and subsequent destruction of the blood-retinal barrier (B S Cummings, 2000; Gong et al., 2014; Giurdanella et al., 2020), so the reduction of cPLA2 phosphorylation provides a basis for DAPA to affect arachidonic acid. Studies have shown that extracellular signal-regulated kinase 1/2 (ERK1/2) is associated with protein-serine/threonine kinases (Roskoski, 2012). Phosphorylated ERK1/2 promotes the phosphorylation of cPLA2 and increases the activity of cPLA2, thereby causing endothelial cell dysfunction (Zhou et al., 2003). Studies have shown that the ERK1/2/cPLA2 axis does an indispensable role in the dysfunction of endothelial cells (Cao et al., 2019). In our experiments, we found that dapagliflozin can reduce the phosphorylation of ERK1/2 in HRMECs caused by high glucose, and provides new theoretical support to understand the anti-apoptotic mechanism of DAPA independent of hypoglycemia. The activity of cPLA2 is not only regulated by ERK1/2 but also directly regulated by intracellular ion levels. Studies have shown that DAPA affects intracellular ion levels. In addition, a new study suggests that VEGF-A participates in hyperglycemia-induced retinal ECs injury by activating the ERK1/2/PLA2 axis (Giurdanella et al., 2020). Whether DAPA affects the activity of ERK1/2/cPLA2 by promoting the release of VEGF-A or changing the intracellular ion level needs further exploration.

Our experiment proposed for the first time that dapagliflozin exerted a protective effect on the diabetic retina by mitigating apoptosis. We innovatively discovered that dapagliflozin affected the release of arachidonic acid in HRMECs and dapagliflozin does not affect glucose uptake. In terms of molecular mechanism, we proposed that the possible mechanism for dapagliflozin to reduce apoptosis was ERK1/2/cPLA2/AA/ROS. Our findings provided a possible molecular mechanism for dapagliflozin to protect DR and provided the basis for dapagliflozin as a drug that can lower glucose and treat DR except for anti-VEGF and laser.

## Conclusions

**FIGURE 7:**
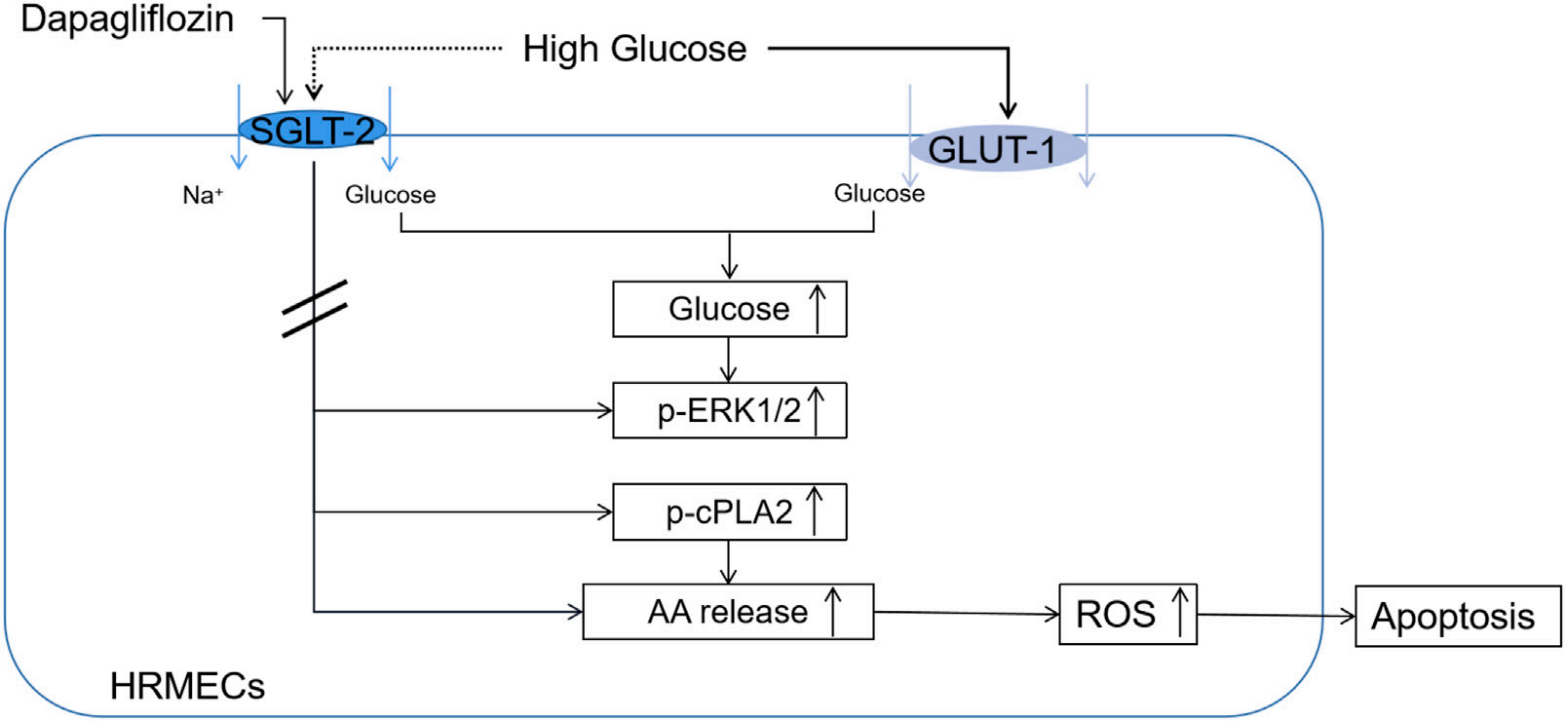
Mechanism of DAPA acting on HRMECs.

In summary, our results indicated that dapagliflozin could mitigate apoptosis of diabetic retina and HRMECs which was independent of hypoglycemic. Dapagliflozin reduced the production of ROS through ERK1/2/cPLA2/AA pathway, thereby alleviating apoptosis of HRMECs **(** Figure 7).

## Data Availability

The original contributions presented in the study are included in the article/Supplementary Material, further inquiries can be directed to the corresponding author.
