# Supplementary figures and images for: Dapagliflozin Reduces Apoptosis of Diabetic Retina and Human Retinal Microvascular Endothelial Cells Through ERK1/2/cPLA2/AA/ROS Pathway Independent of Hypoglycemic
Source: Front Pharmacol. 2022 Feb 24;13:827896. doi: 10.3389/fphar.2022.827896 (PMC8908030; doi:10.3389/fphar.2022.827896)

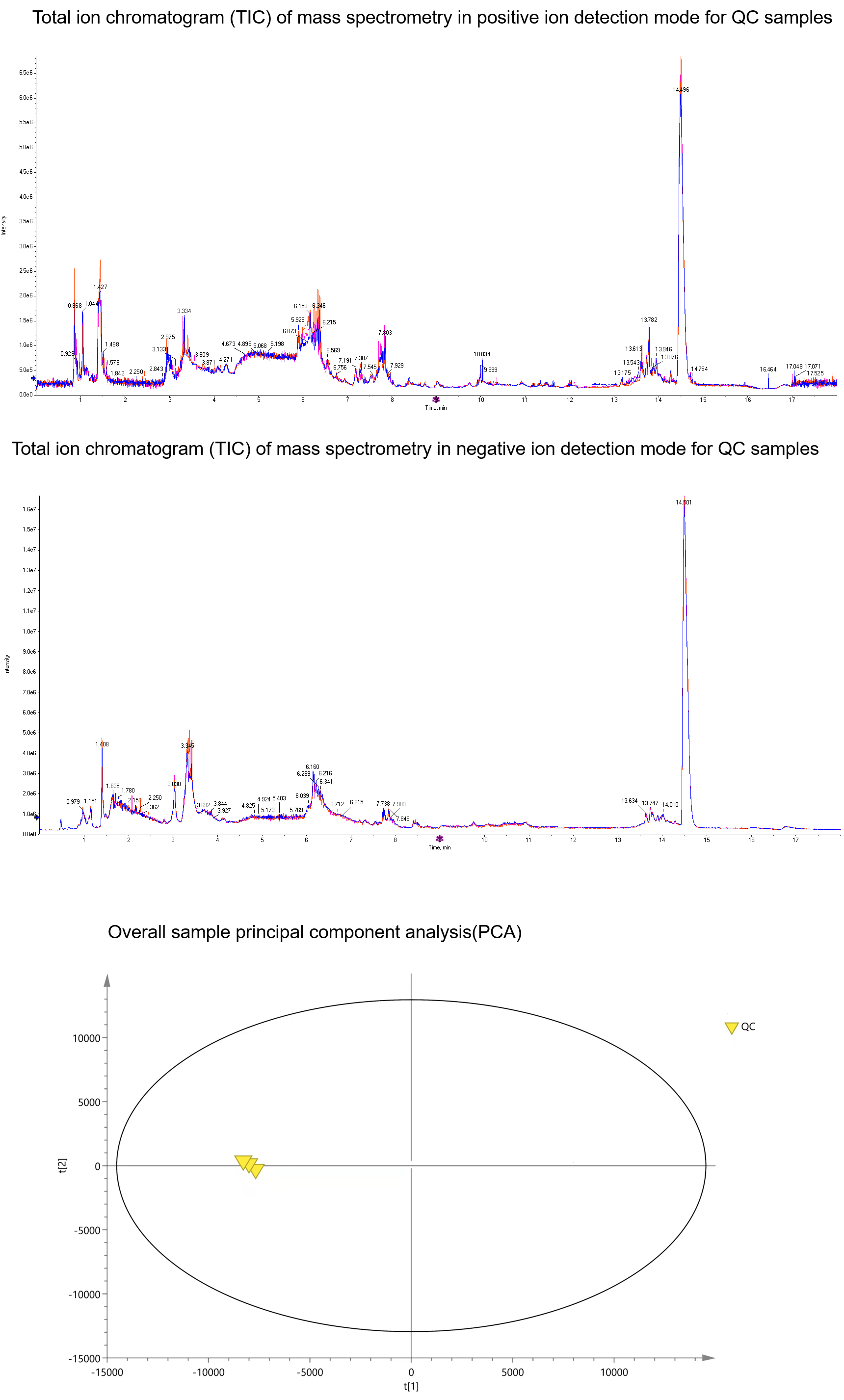

Supplement: Supplementary file 1 [file DataSheet1.ZIP › Supplementary material 1.png]
